# Tracking Demographic Movements and Immunization Status to Improve Children’s Access to Immunization: Field-Based Randomized Controlled Trial

**DOI:** 10.2196/32213

**Published:** 2022-03-01

**Authors:** Jérôme Ateudjieu, Ketina Hirma Tchio-Nighie, André Pascal Goura, Martin Yakum Ndinakie, Miltiade Dieffi Tchifou, Lapia Amada, Marcelin Tsafack, Frank Forex Kiadjieu Dieumo, Etienne Guenou, Charlette Nangue, Bruno Kenfack

**Affiliations:** 1 Department of Health Research Meilleur Accès Aux Soins de Santé (M.A.SANTE) Yaounde Cameroon; 2 Department of Public Health Faculty of Medecine and Pharmaceutical Sciences University of Dschang Dschang Cameroon; 3 Division of Health Operations Research Cameroon Ministry of Public Health Yaounde Cameroon; 4 Department of Microbiology Faculty of Sciences University of Buea Buea Cameroon; 5 University Teaching Hospital Yaounde Cameroon; 6 Department of Gynecology and Obstetrics Faculty of Medicine and Pharmaceutical Sciences University of Dschang Dschang Cameroon; 7 Department of Gynecology and Obstetrics Dschang District Hospital West Cameroon

**Keywords:** immunization status, coverage, completeness, timeliness, EPI vaccines, children under five, Foumban, Cameroon, mobile phone

## Abstract

**Background:**

Countries’ Expanded Program on Immunization (EPI) contribute to the reduction of mortality and morbidity, but access to these vaccines remains limited in most low-income countries.

**Objective:**

We aim to assess whether involving community volunteers (CVs) to track children’s vaccination status and demographic movements and using recorded data to plan catch-up immunization sessions can improve children’s vaccination timeliness, completeness, and coverage.

**Methods:**

This was a field-based randomized controlled trial and communities of the Foumban health district in West Cameroon were allocated to intervention or control groups. In the intervention group, a CV per community was trained to visit households monthly for a year to assess and record in a register, details of EPI-targeted children, their demographic movements and immunization status. The scanned recorded pages were sent to the health center immunization team through WhatsApp and used to organize monthly community catch-up immunization sessions. In the control group, EPI vaccination sessions were routinely conducted. Surveys were conducted at 6 and 12 months from the beginning of the intervention in both study groups to assess and compare immunization timeliness, coverage, and completeness.

**Results:**

Overall, 30 buildings per cluster were surveyed at midline and endline. Of the 633 and 729 visited households in the intervention group at midline and endline, 630 (99.5%) and 718 (98.4%), respectively, consented to participate. In the control group, 507 and 651 households were visited and 505 (99.6%) and 636 (97.7%), respectively, consented to participate. At 12 months intervention, the month one timeliness of bacille Calmette–Guerin (BCG) vaccine did not increase in the intervention group compared with the control group for the age groups 0-11 months (adjusted odds ratio [aOR] 1.1, 95% CI 0.7-1.8) and 0-59 months (aOR 1.1, 95% CI 0.9-1.4), and significantly increased for the first-year BCG vaccine administration for the age group 0-23 months (aOR 1.5, 95% CI 1.1-2.2). The coverage of diphtheria-pertussis-tetanus and hepatitis B+Hemophilus influenzae type B (DPT-Hi +Hb) dose 3 (aOR 2.0, 95% CI 1.5-2.7) and of DPT-Hi+Hb dose 1 (aOR 1.8, 95% CI 1.4-2.4) vaccines increased significantly in the intervention group compared with the control group in the age groups 12-59 months and 12-23 months, respectively. Specific (DPT-Hi+Hb dose 1 to DPT-Hi+Hb dose 3: aOR 1.9, 95% CI 1.4-2.6) and general (BCG to measles: aOR 1.5, 95% CI 1.1-2.1) vaccine completeness increased significantly in the intervention group compared with the control group.

**Conclusions:**

Findings support that involving CVs to track children’s vaccination status and demographic movements and using recorded data to plan catch-up immunization sessions improve children’s vaccination timeliness, completeness, and coverage. This strategy should be adopted to improve access to vaccination for EPI target populations and the consistency verified in other contexts.

**Trial Registration:**

Pan African Clinical Trials Registry PACTR201808527428720; https://pactr.samrc.ac.za/TrialDisplay.aspx?TrialID=3548

## Introduction

### Background

The Expanded Program on Immunization (EPI) has successfully contributed to reducing infant morbidity and mortality worldwide. In many contexts, the EPI’s performance in terms of coverage, completeness, and timeliness remains low and associated with outbreaks of vaccine-preventable diseases [1-3]. In Cameroon, 11 vaccines are planned to be administered to children aged 0-11 months under the EPI [4]. These vaccines are routinely offered at health facilities on a scheduled day on a weekly basis or on a monthly basis in communities with limited geographic access to the vaccination health facilities. Because of limited resources at health facilities (human, financial, vaccine supply and cold chain infrastructure, transportation, and power supply) on the one hand and false perceptions of vaccination, poor information and knowledge of caregivers, and demographic movements of the population on the other hand, many children fail to receive their planned vaccine doses or be vaccinated on time or complete their vaccination schedule as required by the national EPI [1,5].

The 2018 Demographic Health Survey conducted at the household level reported 86.7%, 71.5%, and 65.3% vaccination coverage for bacille Calmette–Guerin (BCG), diphtheria-pertussis-tetanus and hepatitis B+Hemophilus influenzae type B3 (DPT-Hi+HB3), and first-dose measles–rubella vaccines, respectively, among children aged 12-23 months, with a zero-dose proportion of 9.7% [6]. These performances are far below the objectives of the EPI in Cameroon [7]. Many other studies and reports have highlighted heterogeneous immunization coverage rates in Cameroon and a high dropout rate in children’s vaccination cascade. Low vaccination coverage as well as poor timeliness and completeness rates have been reported to be associated with a high incidence of EPI vaccine–preventable diseases [8,9]. Poor maternal socioeconomic status, failing to remember the vaccination schedule, limited access to health care services, below par population health care–seeking behaviors, false perceptions of vaccination, misestimating the targeted population, migrations, and demographic movements are the most cited factors contributing to the low immunization coverage and incomplete vaccination schedule among children [10].

Strategies have been tested in many countries to reduce missed opportunities of vaccination and improve access to vaccines. The strategies frequently reported to have shown some positive impact include providing information on immunization to parents and community members, distributing memory cards specifically designed to help remember immunization schedules, offering vaccines through proximity vaccination sessions with or without incentives, identifying unvaccinated children during home visits and referrals to health facilities, and integration of immunization services within other services [11,12].

### Rationale

During previous EPI supervision activities, we observed that many children and pregnant women miss out on vaccinations during scheduled periods because of short- or long-term travel. In the national immunization guidelines, no procedure has been planned to catch up and reduce the time gaps between the recommended vaccination dates and the dates of vaccine administration. In approximately one-third of the 191 currently functional health districts, most deliveries occur in communities and newborns are not brought into contact with health facilities and thus not considered when planning outreach vaccination sessions. Similarly, periodic trips of caregivers with children as well as immigrants and emigrants are not taken into account when planning or monitoring health facility or outreach immunization sessions. Nomads move constantly from one village to another and are not targeted by immunization sessions. In some cases, nomads’ children receive several doses of the same vaccine at any time on their travels, but none is recorded. This often leads to delaying vaccinating or not vaccinating approximately 30% to 70% of the EPI target population depending on the health district. We assume that a periodic and systematic tracking of children who missed either the timing or ≥1 doses of immunization because of the demographic movement of the parents or limited geographic access to immunization sites and organizing adequate catch-up immunization sessions could significantly improve these children’s immunization coverage, timeliness, and completeness. The aim of this project is to test whether using community volunteers (CVs) to record vaccination status and demographic movements of children at the household level and using the recorded data to plan immunization sessions and catch-up sessions for children missing out on vaccination can improve EPI vaccination timeliness, completeness, and coverage.

## Methods

### Trial Ethical Approval and Registration

The protocol was evaluated and approved by the Cameroon National Ethics Committee for Human Health Research (2018/07/1058/CE/CNERSH/SP), authorized by the Cameroon Ministry of Public Health (631-19-18), and registered with the Pan African Clinical Trials Registry (PACTR201808527428720) on August 22, 2020. Before participation in this study, all heads of households were informed about the survey, and their consent was required before any data were collected in the household. Any data that could reveal the identity of participants were coded and access limited to study members.

### Trial Design

This was a cluster randomized controlled trial in which communities of the targeted health district were randomly assigned to either the intervention group or the control group. In the intervention group, CVs were trained to visit households monthly to record children’s immunization status and demographic movements in a community register. The recorded pages of the register were scanned and sent to the health facility in charge of vaccination for the planning of outreach vaccination sessions in the communities in need. For the control group, EPI vaccination was organized as per routine, meaning on a weekly basis at health facilities or on a monthly basis for outreach activities in communities with limited access to vaccination sites. Community-based surveys were conducted among the intervention and control groups 6 and 12 months after the beginning of the intervention to assess and compare vaccination coverage, timeliness, and completeness rates.

### Study Site and Period

**Figure 1:**
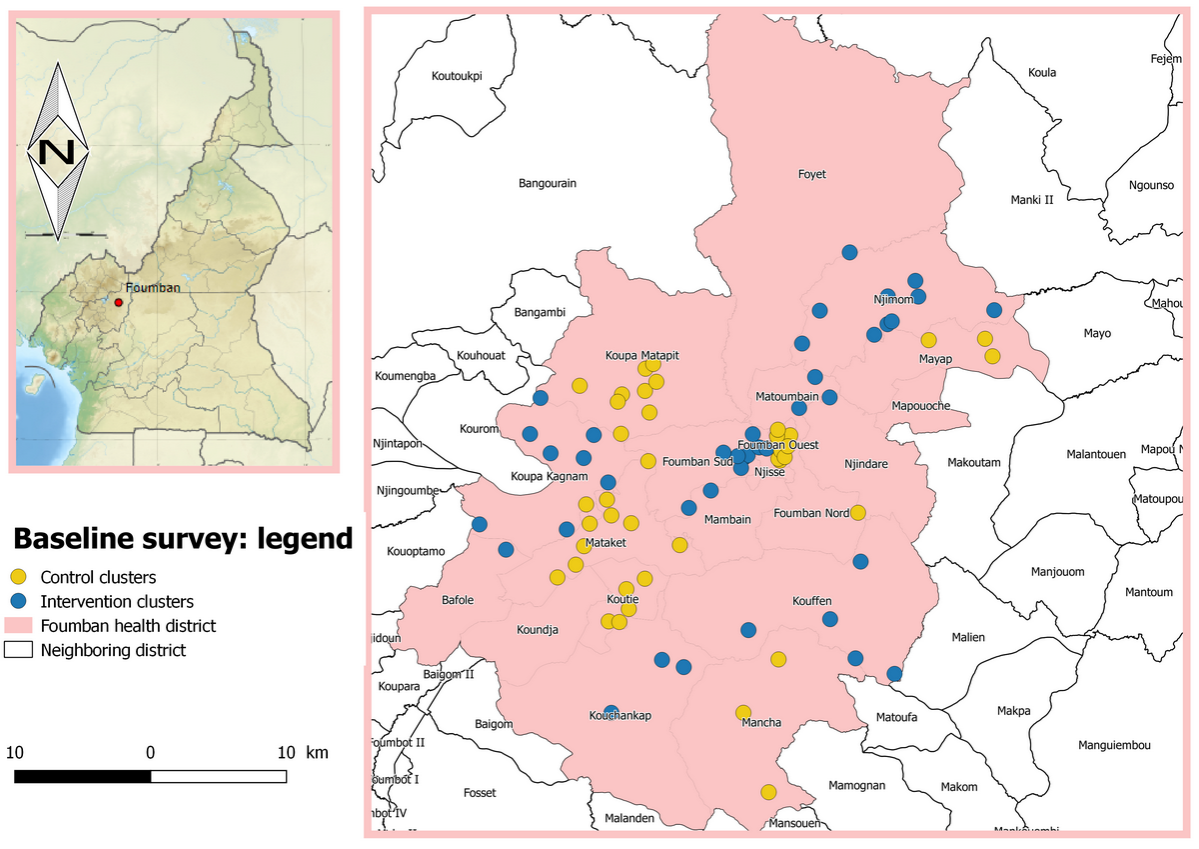
Map of the Foumban health district: clusters involved in the baseline survey.

**Figure 2:**
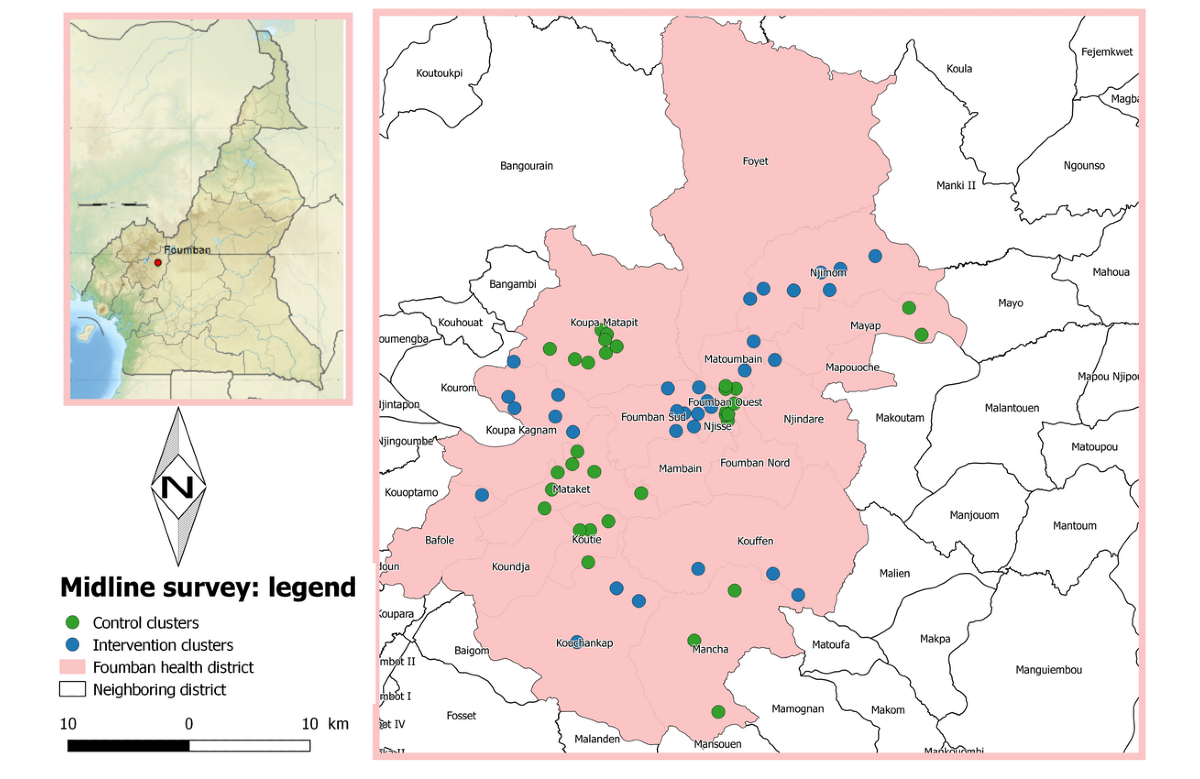
Map of the Foumban health district: clusters of the midline survey.

**Figure 3:**
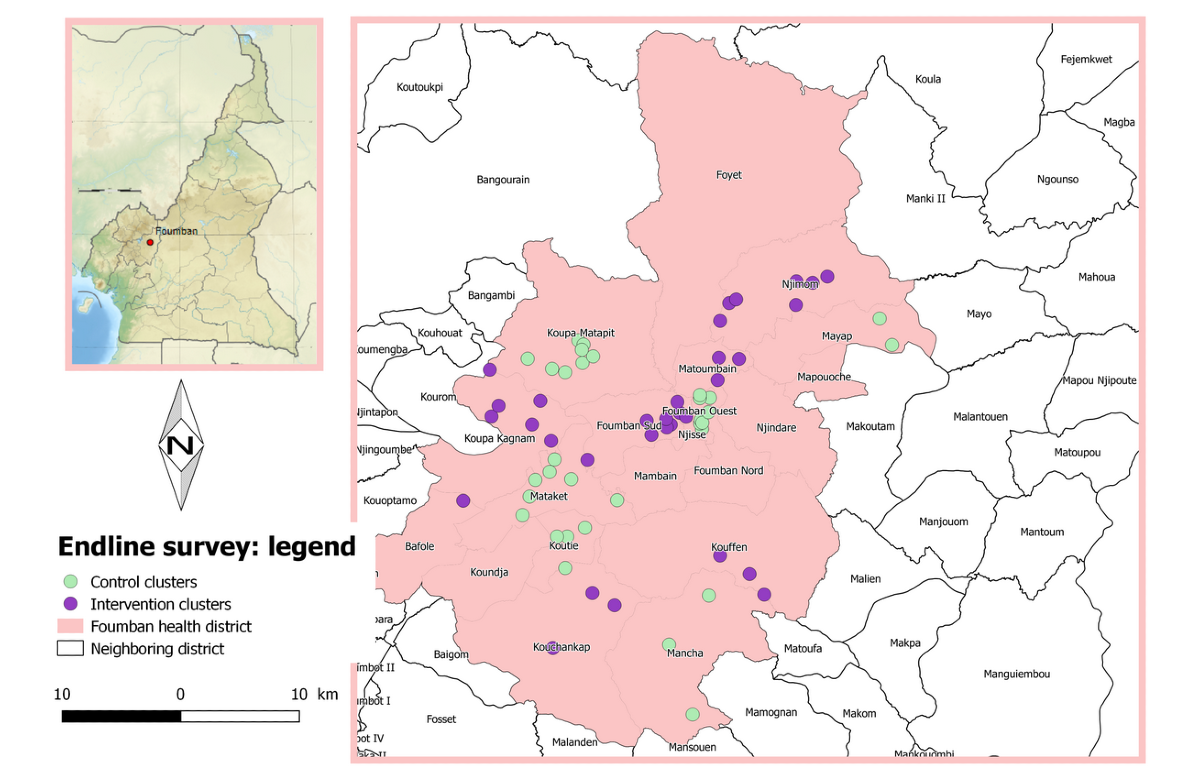
Map of the Foumban health district: clusters involved in the endline survey.

The field phase of this study was conducted from mid-2018 to the third quarter of 2019 in the Foumban health district, West Region, Cameroon. This district is inhabited by seminomadic people who move periodically each year with part of, or all, their household and cattle in search of pasture or for farming activities. From weekly reports of the Epidemiological Surveillance Unit of the Department for the Control of Disease, Epidemics, and Pandemics, Cameroon Ministry of Public Health, it was found that Foumban is one of the health districts that has been affected by at least one outbreak of an EPI vaccine–preventable disease during each of the 5 previous years. Figures 1, 2, and 3 present the clusters involved in the baseline, midline, and endline surveys, respectively.

### Sampling and Randomization of Clusters

In this study, we considered a *community* to be the smallest geographic area (quarter) with a traditional leader (commonly called head of quarter) gathering 100-300 households in rural areas or 200-500 households in urban areas. The list of communities was obtained from the heads of health areas. Communities with limited access to a vaccination health facility and either having a poor vaccination performance (administrative DPT-Hi+HB3 vaccination coverage ˂70% in the routine EPI target population) or having recorded a confirmed case of an EPI vaccine–preventable disease in the previous year (2017) were included. Communities with limited seasonal accessibility limiting the monitoring of the intervention during some period of the implementation of the intervention were excluded.

Selected communities were stratified according to their setting (urban or rural), the importance of yearly population movements, the distance to the vaccination health facility, and the occurrence of EPI vaccine–preventable diseases in the previous year. In each stratum, communities were ranked in alphabetic order from A to Z and in blocks of 2. All combinations of blocks were listed and a single-digit number assigned to each combination. Numbers were generated from Table XXXIII of Yates and Fisher [13] as follows: an arbitrary point was chosen in the table and numbers read in a single-digit row by row across the page. Each number read and corresponding to a pair of communities dictated the distribution of these communities by study group. Each community was divided into subunits of up to 200 buildings called clusters using the Google Maps app installed on a smartphone. From previous experience, this is the number of buildings that can be visited in a week by a CV to implement planned activities. One of these clusters per village was randomly chosen per community and targeted to receive the study intervention.

### Participants

All children aged 0-59 months living in households of the selected clusters were eligible. This included the age group targeted by the Cameroon national EPI for routine immunization (0-11 months) and the catch-up vaccination group program (12-59 months) [4]. Children arriving in a household to stay for less than a month were excluded, and those leaving or planning to stay out of the household for more than a month were also excluded. Those leaving the household for less than a month were included. Parents of children leaving the household to stay away for more than a month were followed up on telephone, when possible, to sensitize them to the necessity of completing the child’s vaccination program.

### Intervention

In each community, a CV was proposed by the head nurse of the competent health center and trained to visit households of the cluster monthly and record in a register all children aged 0-59 months and their demographic movements for the previous and next months and assess their immunization status from the vaccination card or by using a tracking grid if the child did not have a vaccination card. The CVs were from, and inhabitants of, the targeted communities and able to read at least one official language as well as speak the local language. They were persons usually employed by the health system for community interventions (eg, vaccination campaigns). The recorded information page was scanned each month after the visit of all targeted households of a CV’s community and sent through WhatsApp to the immunization team of the competent health center. The information was used by the vaccination team that has received standardized training on reading and using WhatsApp images to plan and implement monthly community immunization sessions. This community vaccination session was conducted with the collaboration of the CVs who choose an accessible vaccination site in the community and inform parents with children needing vaccinations about the session. The activities of vaccination teams and CVs were supervised monthly.

### Control

In the control group, immunization sessions were conducted as per routine. This meant the organization of weekly vaccination sessions by the vaccination team at health facilities and, when possible, monthly vaccination sessions in communities lacking geographic access to the vaccination facilities.

### Outcomes Assessment

Data to assess effects of the intervention were collected using baseline, midline, and endline surveys. The baseline survey was conducted before the intervention, the midline survey was conducted 6 months after the beginning of the intervention, and the endline survey was conducted at the end of the implementation period. Each survey lasted for a week. The baseline survey also provided data on population characteristics and children’s access to EPI vaccination before the intervention. Each cluster was mapped using the *My position* function of the Google Earth smartphone app and divided into subclusters of approximately 30 buildings, assuming that each cluster had at least 20 children aged 0-59 months (as determined from a pretest conducted in the area). One subcluster was randomly selected per cluster and all its households visited for data collection. Data were collected by trained and supervised surveyors using immunization cards, the community immunization register, and questionnaires administered to parents of children. The primary data collected included the immunization status and time regarding the administration of the BCG, polio zero, DPT-Hi+HB1, DPT-Hi+HB2, and DPT-Hi+HB3 vaccines, as well as sociodemographic characteristics. The sampling and implementation processes of the baseline, midline, and endline surveys were similar but independent. The surveys were conducted by independent survey teams different from the team in charge of implementation of the intervention under investigation.

The primary outcome was the documented children’s immunization timeliness, defined as the proportion of children aged <5 years with documented BCG vaccine administration in the first month of life.

The secondary outcomes included the following:

- Documented general EPI-vaccine completeness of children aged 12-59 months, defined as the proportion of children who started the vaccination schedule with the BCG vaccine and completed it by receiving the measles–rubella vaccine, as documented in the immunization card.
- Documented specific immunization completeness of children aged 12-59 months, defined as the proportion of children who received the DPT-Hi+HB1 vaccine and completed pentavalent vaccination doses by receiving the DPT-Hi+HB3 vaccine, as documented in the immunization card.
- Overall children’s immunization timeliness, defined as the proportion of children completing all their EPI-recommended vaccines within the first year of life, as documented in the immunization card or not documented but tracked from the caregiver’s memory.
- Overall children’s immunization completeness, defined as the proportion of children who started the vaccination schedule with the BCG vaccine and completed it by receiving the measles–rubella vaccine, as documented in the immunization card or not documented but tracked from the caregiver’s memory.
- Documented children’s immunization coverage, defined as the proportion of children who received the DPT-Hi+HB3 vaccine, as documented in the immunization card.
- Overall children’s immunization coverage, defined as the proportion of children who received the DPT-Hi+HB3 vaccine, as documented in the immunization card or tracked from the caregiver’s memory.
- Documented recruitment rate, defined as the proportion of children starting the vaccination schedule with the BCG vaccine, as documented in the immunization card.
- Overall recruitment rate, defined as the proportion of children starting the vaccination schedule with the BCG vaccine, as documented in the immunization card or tracked from the caregiver’s memory.

The effects of the intervention were assessed by comparing completeness, timeliness, and coverage rates estimated from the intervention and control groups.

### Sample Size Estimate

Using Stata software (version 16.0 IC; StataCorp LLC), we estimated that the minimum number of children required to test the intervention was 20 children aged <5 years per cluster in at least 23 clusters of the control group and at least 20 clusters in the intervention group. The estimate assumes between-cluster coefficients of variation of 0.38 and 0.19 in the control and intervention groups, respectively (estimated from baseline surveys in clusters assigned to each group), to reach 20 children aged <5 years per cluster in each group, *α* of .05, and 90% power to detect a 10% 2-sided variation of the BCG vaccination timeliness (defined as the proportion of children aged <5 years with documented BCG vaccine administration in the first month of life). The estimate was guided by the method of estimating cluster randomized controlled trials proposed by Batistatou et al [14]. We adjusted the sample size to 32 clusters of at least 20 children per cluster per study group, assuming that 10% of the targeted children would be unreachable (nonresponse and absence during the survey week) and to ensure sufficient power to prevent cluster variation in the estimated outcomes.

### Data Analysis

The effect of the intervention was assessed by estimating per study group, and comparing, the following: (1) yearly immunization timeliness rates for the BCG vaccine (proportion of children aged 0-59 months with evidence of vaccination in the first month of life) and the measles–rubella vaccine (proportion of children aged 12-23 months with evidence of vaccination when aged 9-11 months); (2) the coverage of the BCG vaccine (proportion of children aged 0-59 months who were vaccinated) and the DPT-Hi+HB1, DPT-Hi+HB3, and measles–rubella vaccines (proportion of children aged 12-59 months who were vaccinated); and (3) the specific completeness (proportion of children who completed the DPT-Hi+HB1 and DPT-Hi+HB3 vaccines) and general completeness (proportion of children who completed the BCG and measles–rubella vaccines) when aged 12-59 months. Odds ratios for children being vaccinated, being vaccinated on time, and completing vaccination were estimated and adjusted for the child’s place of birth, guardians’ level of education, type of population (seminomadic or sedentary), profession, walking time to the vaccination site, and religion and controlled for variability of the child’s age using logistic regression random effect. Data were collected using Open Data Kit–designed forms on smartphones, verified in the field, and submitted to a secure server. Data were monitored and cleaned in Microsoft Excel 2013 and analyzed using Stata software (version 16.0 IC).

### Survey Procedures

GPS coordinates were collected at the limits of each selected cluster to map the area and retrieve the map on Google Earth. With the help of CVs, each cluster was divided into multiple subclusters of approximately 30 buildings each. One subcluster was randomly selected, and all the inhabited households of the buildings in this subcluster were visited. All heads of households were informed by the survey team about the project, and only households of those consenting were enrolled. In these households, immunization status data of all children aged 0-59 months were collected from the caregiver and from their immunization cards, along with information on any demographic movement in the previous 6 months. Closed households were visited up to 3 times on 3 different days before being classified as closed. The study team arranged appointments with heads of households and the children’s guardian if they were busy on the first day of the visit. Heads of households and guardians who could not be met after 3 appointments on 3 different days were considered nonrespondents. Children with caregivers refusing to respond to the questionnaires as well as children normally living in the household but absent during the data collection period were excluded.

The study questionnaires were pretested and developed into electronic forms by the data management team. Skip patterns as well as required and formatted fields were used to ensure data accuracy and completeness. Data were collected by trained surveyor teams with smartphones using the Open Data Kit Collect app. Each team of 3 surveyors was trained on the study procedures and supervised daily for participant sampling, informed consent, and data collection processes. A protected web server was deployed by the data management team to compile the survey data. During the survey, completed forms were uploaded to the server daily by the supervisor after reviewing and correcting discrepancies. The data management team ensured daily data cleaning; shared reports with field supervisors; and monitored corrections, updates, and backups. These procedures were the same for both study groups for the baseline, midline, and endline surveys.

### Ethical Consideration

The aim of this study is to test an intervention expected to improve timely access of children to EPI vaccines in areas where children have limited access to vaccination. It involved interacting with communities, heads of households, and caregivers to collect data on children’s vaccination status and demographic movements and organizing vaccination catch-up sessions. All local health, administrative, and traditional authorities with jurisdiction over the targeted study areas were visited and informed about the study and their permission obtained before the implementation of this study. All caregivers were fully informed about the study and provided consent for their participation and that of their children before being included in the study. Surveillance of adverse events was conducted routinely by the health facilities in charge. Data collected in registers for the monitoring of children’s vaccination status were shared between the CVs and the health facility vaccination team, but data extracted from these registers for the study purpose were anonymous and stored in a secure database with access limited to members of the study team. The results and recommendations of the study were presented to representatives of targeted communities, CVs, local and ministerial health authorities, and funders.

## Results

### Participants and Participant Flow

**Figure 4:**
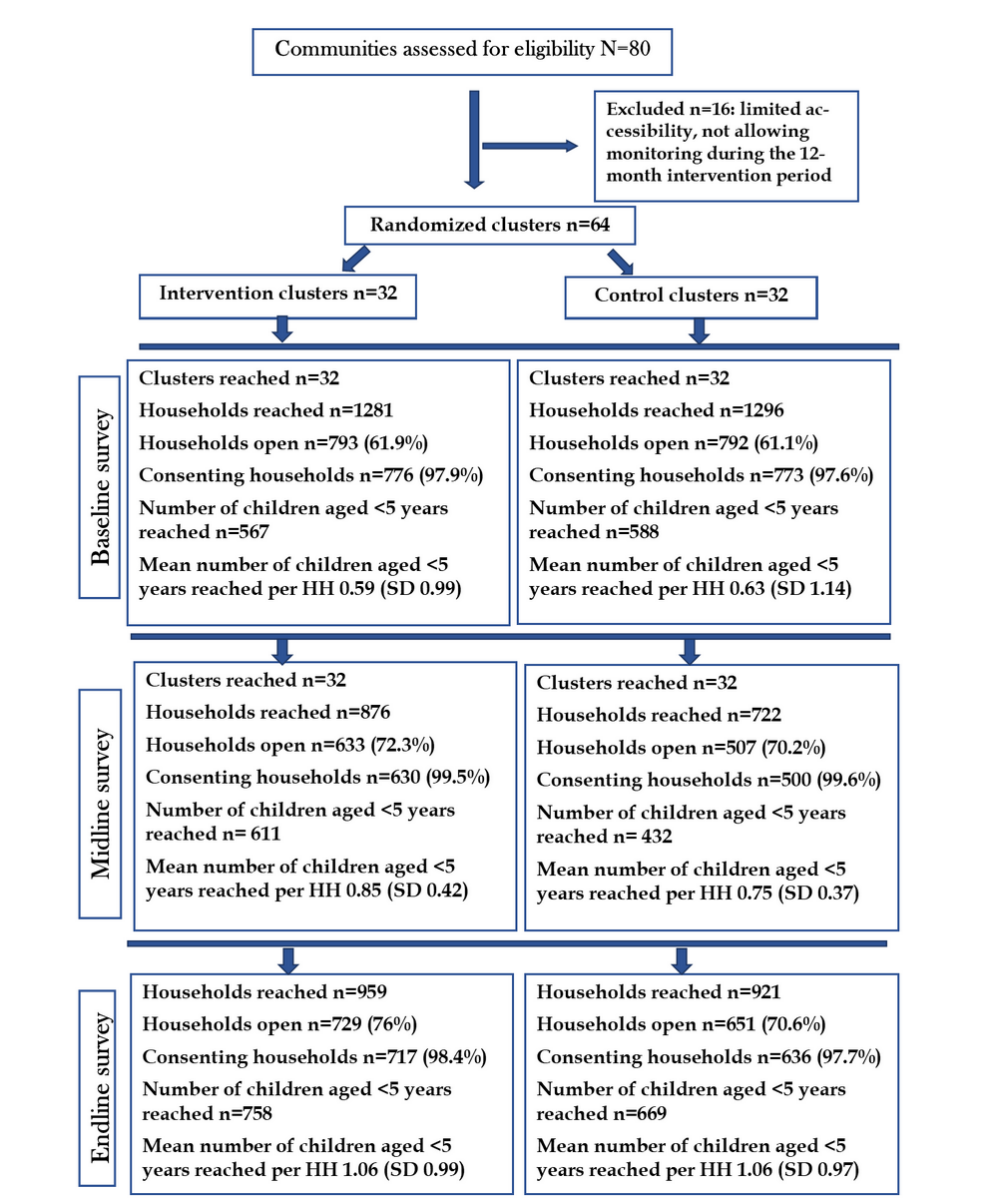
CONSORT (Consolidated Standards of Reporting Trials) flowchart of the study. HH: household.

Clusters from 80 communities in Foumban health district were selected and assessed for eligibility. Of the 80 clusters, 16 (20%) were excluded because they were not accessible enough to facilitate the monitoring of activities all through the year and 64 (80%) were included. Of these 64 clusters, 32 (50%) each were randomly assigned to the intervention and control groups. The mean numbers of buildings, households, and children aged 0-5 years per cluster for both study groups were 161.0 (SD 49.9; 95% CI 149.9-172.1), 119.2 (SD 36.9; 95% CI 109.3-129.0), and 89.7 (SD 36.1; 95% CI 81.7-99.2), respectively. Figure 4 shows the CONSORT (Consolidated Standards of Reporting Trials) flowchart presenting the distribution of visited and consenting households and mean number of children aged <5 years in subclusters selected from the clusters assigned to the intervention and control groups during the midline and endline surveys conducted to assess the effects of the intervention.

### Baseline Characteristics of Study Arms

Baseline characteristics of communities with access to a vaccinating health facility, household and children’s guardians are presented in Table 1. In each of these communities, clusters with a mean of 160.1 (SD 52.5; 95% CI 143.3-176.9) buildings were assigned to the intervention group and clusters with a mean of 161.4 (SD 47.7; 95% CI 146.6-177.2) buildings were assigned to the control group.

**Table 1:** Baseline characteristics of households (HHs) and children’s guardians in the control and intervention study groups.

Variable		Control			Intervention			Chi-square (*df*)		*P* value
		Total	Value, n (%)	Total		Value, n (%)				
**HHs**										
	HHs with approachable road accessible all year round	1295	1290 (99.6)	1281		1280 (99.9)	1.5 (2)		.22	
	Walking time from HH to health center <1 hour	1295	817 (63.1)	1281		584 (45.6)	79.5 (2)		<.001	
	HHs hosting seminomadic populations	1092	584 (18.6)	1012		269 (26.6)	12.2 (2)		<.001	
	Farming as main source of income in the HH	776	231 (29.8)	773		233 (30.1)	0 (2)		.87	
	HHs with uncemented floor	776	320 (41.2)	773		297 (38.4)	1.3 (2)		.26	
**Characteristics of children’s guardians**										
	Woman as child’s guardian	588	556 (94.9)	567		531 (93.7)	0.8 (2)		.36	
	Adolescent as child’s guardian^a^	583	46 (7.9)	563		43 (7.6)	0 (2)		.87	
	Noneducated guardians	588	58 (6.3)	567		100 (17.6)	14.8 (2)		<.001	
	Muslim guardians	588	503 (85.5)	567		504 (88.9)	2.9 (2)		.09	
	Unemployed guardians	588	358 (60.9)	567		341 (60.1)	0.1 (2)		.91	

^a^Of the 1155 respondents, 1146 (99.22%) answered the question about the age of the child’s guardian.

### Baseline Characteristics of Clusters, Households, and Participants in Both Study Groups

Of the 32 clusters in each study group, 8 (33%) were urban clusters and 24 (67%) were rural clusters. Of the 32 clusters in each study group, the control group had 5 (16%) with mainly seminomadic populations, whereas the intervention group had 3 (9%; Table 1). The difference between the groups was not significant (Fisher exact test=0.708; dof=2).

### Baseline Documented Vaccination Coverage per Study Arm

The distribution of baseline vaccination coverage per study arm is presented in Table 2. Vaccination coverage regarding the BCG vaccine, which is the first vaccine administered, the DPT-Hi+HB1-3 vaccines, which are the main indicators of EPI performance, and the measles–rubella vaccine was not different at baseline between the study groups.

**Table 2:** Baseline documented (with vaccination card) vaccination coverage in the clusters allocated to the control and intervention groups.

Vaccine	Vaccination coverage in clusters								
	Control			Intervention			Chi-square (*df*)		*P* value
	Total	Value, n (%)	Total		Value, n (%)				
BCG^a^ coverage among children aged 0-59 months	588	169 (28.7)	567		183 (32.3)	2.8 (2)		.25	
BCG vaccination within the first months of life among children aged <5 years	588	148 (25.2)	567		158 (27.9)	1.2 (2)		.29	
BCG vaccination within the first months of life among children aged 0-11 months	144	60 (41.7)	140		66 (47.1)	0.9 (2)		.35	
BCG vaccination within the first 12 months of life among children aged <5 years	588	165 (28)	567		175 (30.9)	1.1 (2)		.29	
BCG vaccination within the first 12 months of life among children aged 0-23 months	270	104 (38.5)	254		105 (42.1)	0.7 (2)		.40	
Pentavalent 1 coverage among children aged 12-59 months	427	104 (24.4)	444		96 (21.6)	0.9 (2)		.34	
Pentavalent 3 coverage among children aged 12-59 months	427	82 (19.2)	444		85 (19.1)	0 (2)		.98	
Pentavalent 3 coverage among children aged 12-23 months	114	27 (23.7)	126		31 (24.6)	0 (2)		.87	
MR^b^ coverage among children aged 12-59 months	427	68 (15.9)	444		58 (13.1)	1.4 (2)		.23	
MR vaccination within the first 9-11 months of life among children aged 12-59 months	427	55 (12.9)	444		49 (11)	0.7 (2)		.40	
Specific completeness (DPT-Hi+HB1-3^c^)	427	80 (18.7)	444		85 (19.1)	0(2)		.88	
General completeness (BCG-MR)	427	58 (10.7)	444		58 (13.1)	1.4 (2)		.23	

^a^BCG: bacille Calmette–Guerin.

^b^MR: measles–rubella.

^c^DPT-Hi+HB: diphtheria-pertussis-tetanus and hepatitis B+Hemophilus influenzae type B.

### Flow per Study Arm of Some Characteristics During the Study Period

The distribution per study arm of the characteristics presented in Table 3 did not differ at baseline, but there were statistical differences regarding some attributes such as the mean number of households visited at least once by a CV during the previous 6 months, mean number of births in the previous 6 months at midline, proportion of open households, mean age of children, number of households visited at least once by a CV during the previous 6 months, and availability of the vaccination card among children aged <5 years at the endline.

**Table 3:** Evolution of some study arm characteristics from baseline to midline and endline surveys.

Characteristics	Baseline survey						Midline survey						Endline survey				
	Intervention	Control	Chi-square (*df*)	*t* test (*df*)	*P* value	Intervention		Control	Chi-square (*df*)	*t* test (*df*)	*P* value	Intervention		Control	Chi-square (*df*)	*t* test (*df*)	*P* value
Proportion of open HHs^a^, n/N (%)	793/1281 (61.9)	792/1296 (61.1)	0.2 (2)	—^b^	.69	633/876 (72.3)		507/722 (70.2)	0.8 (2)	—	.37	729/959 (76)		651/921 (70.6)	6.8 (2)	—	<.001
Proportion of open HHs consenting to participate, n/N (%)	776/793 (97.9)	773/792 (97.6)	0.5 (2)	—	.50	630/633 (99.5)		500/507 (99.6)	2.7 (2)	—	.10	717/729 (98.4)		636/651 (97.7)	0.8 (2)	—	.38
Number of children aged <5 years reached per HH, mean (SD)	0.59 (0.99)	0.63 (1.14)	—	0.93 (2)	.35	0.85 (0.42)		0.75 (0.37)	—	1.67 (2)	.09	1.060 (0.99)		1.057 (0.97)	—	0.054 (2)	.96
Age of children aged <5 years (in months), mean (SD)	26.01 (16.76)	26.16 (16.26)	—	0.18 (2)	.86	24.51 (16.7)		25.55 (16.08)	—	1.003 (2)	.31	27.33 (20.12)		30.0 (20.62)	—	2.48 (2)	.01
Proportion of girls among children, n/N (%)	283/567 (49.9)	307/588 (52.2)	0.3 (2)	—	.60	287/611 (47)		225/432 (52.1)	2.6 (2)	—	.10	371/758 (48.9)		342/669 (51.1)	—	0.312 (2)	.74
Number of HHs visited at least once by a CV^c^ during the previous 6 months, mean (SD)	0.11 (0.48)	0.09 (0.44)	—	0.46 (2)	.65	5.96 (3.64)		2.57 (2.05)	—	18.57 (2)	<.001	5.30 (4.54)		1.77 (1.87)	—	18.19 (2)	<.001
Number of births in the previous 6 months, mean (SD)	0.071 (0.32)	0.074 (0.28)	—	0.19 (2)	.84	0.14 (0.37)		0.10 (0.33)	—	1.965 (2)	.05	0.17 (0.40)		0.16 5 (0.41)	—	0.275 (2)	.78
Number of deaths among children aged <5 years in the previous 6 months, mean (SD)	0.008 (0.10)	0.009 (0.09)	—	0.22 (2)	.82	0.014 (0.13)		0.006 (0.08)	—	1.245 (2)	.21	0.025 (0.19)		0.026 (0.18)	—	0.170 (2)	.87
Number of children aged <5 years who left the HH for at least a month during the previous 6 months, mean (SD)	0.061 (50.35)	0.057 (0.32)	—	0.34 (2)	.83	0.16 (0.71)		0.18 (0.70)	—	0.441 (2)	.65	0.154 (0.59)		0.202 (0.63)	—	1.414 (2)	.16
Number of children aged <5 years who arrived in the HH in the previous 6 months to stay for at least a month, mean (SD)	0.10 (0.48)	0.11 (0.51)	—	0.61 (2)	.54	0.114 (0.19)		0.0701 (0.38)	—	1.65 (2)	.09	0.093 (0.61)		0.091 (0.37)	—	0.082 (2)	.93
Availability of vaccination card among children aged <5 years, n/N (%)	187/567 (33)	173/588 (29.4)	1.8 (2)	—	.18	293/611 (48)		180/432 (41.7)	4.0 (2)	—	.05	643/758 (84.8)		502/669 (75)	—	21.5 (2)	<.001

^a^HH: household.

^b^Not available.

^c^CV: community volunteer.

### Estimated Outcomes of the Intervention for the Evaluation Periods

At midline, the timeliness (proportion of children with BCG vaccination in the first month of life) did not vary, whereas DPT-Hi+HB1 and measles–rubella vaccination coverage in the age group 12-59 months increased significantly in the intervention group compared with the control group. Specific and general vaccine completeness were not different between the control and intervention groups. At endline, the timeliness (proportion of children with BCG vaccination in the first month of life) did not vary, whereas the coverage of all vaccines, apart from the BCG vaccine, in the age group 0-59 months increased significantly as did the specific and general vaccine completeness in the intervention group compared with the control group. Table 4 presents the coverage of vaccines with respect to age groups at midline and endline.

**Table 4:** Outcomes of the intervention at midline and endline.

Vaccine dose	Age group (in months)	Outcomes of midline survey											Outcomes of endline survey								
		Coverage: intervention group			Coverage: control group			aOR^a^ (95% CI)		*P* value		Coverage: intervention group				Coverage: control group			aOR (95% CI)		*P* value
		Total	Value, n (%)	Total		Value, n (%)					Total			Value, n (%)	Total		Value, n (%)				
D^b^: BCG^c^ coverage	0-59	611	382 (62.5)	432		256 (59.3)	0.9 (0.7-1.2)		.34		758			303 (40)	669		231 (34.5)	1.3 (1.0-1.7)		.08	
D: BCG vaccination in the first month of life	0-59	611	186 (30.4)	432		153 (35.4)	0.8 (0.5-1.3)		.34		758			264 (34.8)	669		215 (32.1)	1.1 (0.9-1.4)		.50	
D: BCG vaccination in the first month of life	0-11	167	73 (43.7)	105		64 (61)	0.9 (0.5-1.9)		.84		219			105 (48)	169		78 (46.1)	1.1 (0.7-1.8)		.69	
D: BCG vaccination in the first year of life	0-23	252	107 (42.1)	270		104 (38.5)	1.6 (0.7-3.4)		.51		352			200 (56.8)	276		135 (48.9)	1.5 (1.1-2.2)		.02	
D: DPT-Hi+HB1^d^ coverage	12-59	444	176 (39.7)	327		103 (31.5)	1.5 (1.0-2.0)		.02		539			230 (42.7)	500		143 (28.6)	1.8 (1.4-2.4)		<.001	
D: DPT-Hi+HB3 coverage	12-59	444	149 (33.6)	327		92 (28.1)	1.3 (1.0-1.8)		.10		539			205 (38)	500		117 (23.4)	2.0 (1.5-2.7)		<.001	
D: DPT-Hi+HB3 coverage	12-23	134	57 (42.5)	99		42 (42.3)	1.1 (0.8-1.5)		.64		133			73 (54.9)	107		44 (41.1)	1.7 (1.0-3.0)		.04	
D: Measles–rubella coverage	12-59	444	143 (32.2)	327		74 (22.6)	1.7 (1.2-2.2)		.02		539			214 (39.7)	500		100 (20)	2.6 (1.9-3.5)		<.001	
D: Measles–rubella coverage	12-23	134	49 (36.6)	99		31 (31.3)	1.3 (0.7-2.3)		.37		133			73 (54.9)	107		37 (34.6)	2.3 (1.3-3.9)		.003	
Specific completeness	12-59	444	143 (32.2)	327		89 (27.2)	1.3 (0.9-1.8)		.11		539			196 (36.4)	500		114 (22.8)	1.9 (1.4-2.6)		<.001	
General completeness	12-59	444	106 (23.9)	327		73 (22.3)	1.3 (0.8-1.5)		.49		539			145 (26.9)	500		96 (19.2)	1.5 (1.1-2.1)		<.001	

^a^aOR: adjusted odds ratio.

^b^D: documented.

^c^BCG: bacille Calmette–Guerin.

^d^DPT-Hi+HB: diphtheria-pertussis-tetanus and hepatitis B+Hemophilus influenzae type B.

## Discussion

### Principal Findings

This study assessed the effect of monthly household visits and tracking of vaccination status and demographic movements of children aged <5 years for the planning of community-based catch-up immunization sessions on immunization coverage, timeliness, and completeness. To the best of our knowledge, this is an innovative approach that has not yet been tested. One year after the implementation of the intervention, the timeliness of BCG vaccine administration in the first month of life increased in the intervention group compared with the control group for the age groups 0-11 months and 0-59 months, but the increase was not significant, whereas the increase was significant for the first-year BCG vaccine administration for the age group 0-23 months. The BCG vaccine immunization coverage for the age group 0-59 months increased in the intervention group but not significantly compared with the control group, whereas the increase was significant for the DPT-Hi+HB3 and measles–rubella vaccines for the age groups 12-59 months and 12-23 months. Specific (DPT-Hi+HB1 and DPT-Hi+HB3) and general (BCG–measles–rubella) vaccine completeness increased significantly in the intervention group compared with the control group.

The immunization schedule of the EPI vaccines in a given country is based on local epidemiology and maturity of children’s immune systems [15,16]. In Cameroon, the EPI vaccination calendar is drawn from the document of EPI norms and standard operating procedures [4]. Despite the fact that this calendar is posted at almost all health facilities in the country, very few children receive the EPI vaccines during the recommended period [1]. From the baseline status in which there was no difference in the BCG vaccination timeliness in the intervention group compared with the control group, the BCG vaccine administration in the first year of life improved significantly in the intervention group compared with the control group 1 year after the implementation of the tested intervention. In contrast, the rate for the BCG vaccine administration in the first month of life increased in the intervention group compared with the control group, although the increase was not statistically significant, in the age groups 0-11 months and 0-59 months. The effect of the intervention evaluated in this study has not been evaluated in a previous study to the best of our knowledge. Interventions such as reminders were tested in other settings and were found to not significantly increase the coverage and timeliness of some EPI vaccines [17,18]. The fact that the intervention did not significantly improve the timeliness of BCG vaccine administration at 1 month for the age group 0-59 months was probably because of some reasons such as the following: a large proportion of the evaluated age group had passed the age of eligibility to receive the BCG vaccine given that the Cameroon EPI does not allow catch-up doses of BCG vaccines for children aged ≥1 year [4]. In addition, frequent BCG vaccine stockouts were recorded by vaccination teams, health facilities, and the health district resulting from problems in the supply system and vaccine wastage. To prevent vaccination wastage, most vaccination teams had decided not to open the BCG vial when fewer than 15 children in need were present at a vaccination session. Several appointments were given to reach the minimum number of children before opening the BCG vial, and this delayed the vaccination schedule. Other reasons could have explained the low timeliness rate in the control and intervention groups, but the fact that characteristics were randomized in the 2 study groups could reduce the effect of some of these determinants [19]. Contamination of the control area by the intervention was noted and probably contributed to reducing the difference in the effect of the intervention on vaccine timeliness, and this is supported by the increasing number of visits by CVs to households in the control group from the baseline to midline and endline surveys. The fact that the first-year BCG vaccination coverage in the age group 0-23 months (those who spent part of their first year of life in the project intervention period) was significantly higher in the intervention group than in the control group supports that despite multiple appointments caused by the fear of high vaccine wastage and other obstacles, the intervention was associated with a longer time to induce a significant increase of vaccine timeliness in the intervention group compared with the control group. The effect of the intervention can be considered the only explanation for the higher BCG vaccine coverage in the first year of life in the intervention group as expected, and the identified confounders were used for adjustment when computing the analysis comparing the first-year BCG vaccine timeliness rates in both study groups. These results indicate that even with multiple appointments given by vaccination teams to reduce BCG vaccine wastage and with other barriers preventing a proportion of children from being vaccinated in time, new strategies are needed to significantly improve children’s access to the BCG vaccination in their first month of life; for example, a study could examine whether reducing the number of vaccine doses per BCG vial may be more effective in reducing vaccine wastage, as well as reducing the number of appointments that need to be made, which delays childhood immunization. Another study could examine the contribution of synchronization of BCG vaccination sessions by different vaccination teams in a given district and the mutualization of BCG vials to reduce vaccine wastage and delays. Given that the intervention significantly improved children’s access to BCG vaccination before the end of their first year of life, we believe that it can be recommended in similar contexts as this study and evaluated in other contexts with the hope of using it to improve children’s timely access to BCG vaccination in other contexts in need.

Vaccination coverage determines herd immunity for each targeted disease. The initial situation that existed before the intervention tested in this study could be described as one of low vaccination coverage among children for most EPI vaccines offered [1,6]. The results of the midline and endline surveys conducted 6 months and 12 months, respectively, after the beginning of the intervention show progress, with a significant increase in vaccination coverage in the intervention group for the DPT-Hi+HB3 and measles–rubella vaccines for the age groups 12-23 months and 12-59 months. The fact that there was no significant difference regarding BCG and measles–rubella vaccination coverage between the 2 groups before the intervention, that the assigning of clusters to the intervention and control groups was randomized, and that confounders were adjusted for when comparing vaccination coverage in both study groups support that the intervention contributed to the higher DPT-Hi+HB3 and measles–rubella vaccination coverage in the intervention group. Several studies have pointed out parental and guardian forgetfulness, ignorance, refusal of vaccination, limited geographic access of children to vaccination health facilities, and lack of updated data on vaccine targets because of population movements as factors that limit children’s access to vaccination and contribute to low EPI-vaccine coverage in children [20-22]. The intervention tested in this study proposes periodic household visits to track the immunization status of children and their demographic movements, the organization of community-based immunization catch-up sessions to vaccinate those who need to be vaccinated, and communication with parents and caregivers to convince them to bring these children to the organized vaccination sessions. The intervention thus makes it possible to determine the number of EPI-targeted children living in each community each month, identify those needing each of the EPI vaccinations, use this information to communicate with parents and vaccination teams, and organize immunization sessions at locations and on dates chosen by the community and thus more accessible to the children’s guardians. This is expected to increase immunization coverage, given that it helps to anticipate the main determinants limiting children’s access to EPI vaccines. A given number of interventions targeting health care providers, caregivers, parents or guardians, and communities and testing several strategies such as reaching out (by SMS text messages or telephone calls), home visits, and training have shown varying degrees of effectiveness in improving immunization coverage [12,23,24]. The particularity of the intervention tested in this study is that it combines several approaches tested in previous studies and innovates by involving CVs in the assessment of immunization status and using 2 immunization coverage–monitoring registers, one of which is used by the community to update children’s immunization status, with the updated pages scanned and sent using WhatsApp to the health facility to update the health facility–based register for planning of immunization sessions. The intervention also involves collaboration among households, CVs, and vaccination teams to organize catch-up vaccination sessions. These innovations help to promote the sustainability of our intervention because it is locally organized at a lower cost, it is accepted by the different actors involved in vaccination campaigns, and is therefore expected to be easily scalable across contexts. We recommend that this intervention should be considered an alternative strategy for health systems that plan to address the issue of low EPI vaccination coverage.

The EPI aims to give each cohort of children a package of a certain number of doses of vaccines in their childhood. This package enables each of these cohorts to be protected against vaccine-preventable childhood infectious diseases. A number of studies indicate that vaccine completeness remains quite insufficient for several cohorts of children born in Africa, including in Cameroon [1,5]. As noted with regard to the timeliness and coverage of some key vaccines probably attributable to the intervention tested in this study, the specific (DPT-Hi+HB1 and DPT-Hi+HB3) and general (BCG–measles–rubella) vaccine completeness increased significantly in the intervention group of this study. In addition to the reasons presented in the previous paragraph supporting children’s access to vaccination, the intervention contributed to improving the timeliness because it included repeated household visits to monitor the progress of children’s vaccination status and ensured that they received all doses of the recommended vaccines. This adds to the already discussed benefit of this intervention, which is its ability to act as a tool to monitor single and subsequent vaccine doses recommended by the EPI.

### Limitations

Some limitations were associated with the methodology and implementation of this project. The availability of some vaccines was not assured throughout the project period. This was beyond the control of the study team because supplying vaccines for a routine EPI initiative is the responsibility of the health system and thus was not part of the intervention. The unavailability of vaccines was very heterogeneous in terms of duration, geographic area, and reason but was more frequent and more extensive in terms of duration and geographic area for the BCG vaccine. The reason was the high wastage rate resulting from the high number of doses in the vial compared with the number of children expected to be vaccinated and the obligation to apply the open vial policy. The fact that this was the case for both the intervention and control groups likely contributed to reducing the magnitude of difference in the outcomes between the 2 study groups.

A number of constraints overlapped with the implementation of the project and could contribute to reducing the difference in effect between the intervention group and the control group. Because of the lack of human resources, the health workers in charge of vaccination were unable to attend a certain number of vaccination sessions planned as part of the intervention in the community. The absence of cold chain infrastructure at some health facilities and the periodic unavailability of electricity due to interruptions in electricity supply contributed to reducing the access of the targeted population to the intervention. In addition, the study area has benefited from half-a-dozen campaigns offering a number of interventions to the community and using the vaccination teams and CVs involved in this project, thus contributing to reducing the promptness and coverage of the activities planned in the evaluated intervention through work overlap. Furthermore, the movement of children with their parents or caregivers for agricultural and animal husbandry activities probably contributed to reducing the access of the targeted population to the intervention. Contamination of the control area by the intervention was noted. Both zones (where the study clusters are located) are in charge of routine EPI initiatives, and the heads of the health centers in these zones meet monthly to present and discuss their vaccine performances. During the evaluation surveys, we noted an increase in household visits by volunteers in the control group to implement the project intervention. The evaluation of the effect of the intervention focused on the documented immunization status of children in the households. A child’s immunization record is the best source of data to certify the child's immunization status, but the retention of immunization records by caregivers was not certain and could contribute to underestimating immunization coverage in the intervention and control groups.

These limitations on vaccine availability, cold chains, human resources, contamination, and nonretention of immunization records are part of the context in which the intervention was tested. The fact that the effect of the intervention remains significant for several outcomes in the intervention group suggests that the intervention may improve vaccine timeliness, coverage, and completeness despite these constraints. These results also mean that by ensuring the minimum availability of human resources, cold chain infrastructure, and vaccines, a greater benefit from the intervention can be expected.

### Conclusions

We can conclude from this study that training CVs and organizing and supervising them to ensure monthly household visits to assess the immunization status of children and communicate it to vaccination teams to organize catch-up vaccination sessions increases the timeliness, coverage, and completeness of routine EPI-vaccine administration in the target population. This was illustrated in this study by an increase, although not significant, of first-month BCG vaccine administration timeliness for the age groups 0-11 months and 0-59 months in the intervention group, as well as a significant increase of first-year BCG vaccine administration timeliness for the age group 0-23 months in the intervention group. The coverage of DPT-Hi+HB3 and measles–rubella vaccines for the age groups 12-23 months and 12-59 months as well as the specific (DPT-Hi+HB1 and DPT-Hi+HB3) and general (BCG–measles–rubella) vaccine completeness also illustrated this. In health districts with similar contexts to the one where the intervention was tested, the tested intervention should be proposed to the health system to improve children’s access to EPI vaccines. The efficiency of this intervention should be evaluated in other contexts. For the evaluation and implementation of the intervention, we recommend ensuring the minimum prerequisites for the implementation of the intervention activities, such as the availability of human resources to ensure, when necessary, the immunization activities; the availability of vaccines and cold chain infrastructure; the involvement of a motivated and trained team of supervisors; logistic support for the immunization teams; and the coverage of the shortfall in terms of work by the teams involved when other activities are ongoing. The benefit of this strategy compared with that of the immunization campaign with regard to improving access to immunization and prevention of vaccine-preventable diseases should be assessed.

## Appendix

Multimedia Appendix 1 CONSORT Checklist (V 1.6.1).

## Abbreviations

BCG: bacille Calmette–Guerin
CONSORT: Consolidated Standards of Reporting Trials
CV: community volunteer
DPT-Hi+HB: diphtheria-pertussis-tetanus and hepatitis B+Hemophilus influenzae type B
EPI: Expanded Program on Immunization
